# INTERACTIONS BETWEEN GENES FROM AGING-RELATED PATHWAYS: IMPACT ON HUMAN LONGEVITY

**DOI:** 10.1093/geroni/igz038.3284

**Published:** 2019-11-08

**Authors:** Svetlana Ukraintseva, Matt Duan, Deqing Wu, Konstantin Arbeev, Alexander Kulminski, Joseph M Zmuda, Kaare Christensen, Anatoliy Yashin

**Affiliations:** 1 Duke University, Durham, North Carolina, United States; 4 Department of Epidemiology, University of Pittsburgh; Pittsburgh, Pennsylvania, United States; 5 Danish Aging Research Center, University of Southern Denmark, Odense C, Denmark

## Abstract

Role of genetic interactions (GxG) in human longevity remains poorly understood. We hypothesized that GxG between genes from biologically connected pathways involved in aging may impact longevity. To test this hypothesis, we selected 53 candidate genes from the aging-related pathways (IGF-1/AKT/FOXO3A, TP53/P21/P16, and mTOR/S6K mediated) that are known to jointly influence outcomes of cell responses to stress and damage, such as apoptosis, senescence, growth/proliferation, and autophagy. We evaluated the effects of interactions between SNPs in these genes on longevity in LLFS and CARe data. RESULTS: The IGF1R, PPARGC1A and BCL2 genes were consistently involved in top GxG effects (p<10-6) on survival in the oldest old (85+ and 95+). One SNP, rs2970870 in PPARGC1A gene, was broadly involved in significant interaction effects on survival 96+ (p<10-7) when paired with SNPs in IGF1R and NFKB1 genes. This SNP individually was associated with survival with nominal significance only; therefore, it would have not been selected in a GWAS. We conclude that interactions between genes from aging-related pathways that regulate cell responses and resilience to damage may have major impact on human longevity and contribute to its genetic heterogeneity. The research was supported by the NIA/NIH grants R01AG062623, U19AG063893, P01AG043352.

